# Inflammatory Markers and their Relationship with Cognitive Function in Alzheimer’s Disease and Mild Cognitive Impairment. Systematic Review and Meta-Analysis

**DOI:** 10.1007/s12017-025-08866-w

**Published:** 2025-07-25

**Authors:** Maria Fernanda Serna, Mildrey Mosquera, Herney Andrés García-Perdomo

**Affiliations:** 1 Grupo de Nutrición, Department of Physiological Sciences, University of Valle, Cali, Colombia; 2 Faculty of Health, Universidad Santiago de Cali, Cali, Colombia; 3 Division of Urology/Uro-oncology, Department of Surgery, School of Medicine, Universidad del Valle, Cll 4B # 36-00, Cali, Colombia

**Keywords:** Alzheimer, Biomarkers, Interleukins, Neurosciences

## Abstract

This study estimates the association between blood levels of inflammatory markers and cognitive function in adults with Alzheimer’s disease or mild cognitive impairment. A systematic review and meta-analysis were conducted to synthesize data from studies examining the relationship between blood levels of inflammatory markers and cognitive function in adults with Alzheimer’s disease or mild cognitive impairment. The search strategy was applied to the Medline database through MEDLINE (OVID), WEB OF SCIENCE, SCOPUS, LILACS, and the Cochrane Central Register of Controlled Trials (CENTRAL). Studies were selected based on predefined criteria. We included 84 studies in the qualitative synthesis, with 75 incorporated into the meta-analysis. In blood samples, subjects with Alzheimer’s disease showed significantly higher concentrations of IL-1β (mean difference 0.46 [95% CI: 0.35, 0.58]), IL-6 (3.41 [3.05, 3.78]), MCP-1 (26.20 [14.57, 37.82]), and TNF-α (6.68 [5.97, 7.39]), along with lower concentrations of IL-8 (− 1.46 [− 1.85, − 1.08]) and IL-10 (− 3.20 [− 4.21, − 2.20]). This systematic review and meta-analysis demonstrate significant alterations in inflammatory marker concentrations in blood samples of individuals with Alzheimer’s disease and mild cognitive impairment. Elevated levels of IL-1β, IL-6, MCP-1, and TNF-α, alongside reduced IL-8 and IL-10 levels, suggest a robust inflammatory response associated with Alzheimer’s disease.

**Supplementary Information:**

The online version contains supplementary material available at 10.1007/s12017-025-08866-w.

## Introduction

Alzheimer’s disease is the most common form of dementia globally. Currently, more than 57 million people suffer from some form of dementia worldwide, of which 60% could be attributed to Alzheimer’s disease (AD) (Gustavsson et al., 2023; Song et al., 2023). Alzheimer’s dementia has become one of the leading public health challenges worldwide. It is estimated that over the next 30 years, there will be a 117% increase (Nichols et al., 2022) in cases due to aging, increased life expectancy, and changes in habits that promote the emergence of risk factors (Legdeur et al., 2018).

Neuropathological hallmarks of AD are the accumulation in the brain of plaques composed of beta-amyloid (Aβ) peptide deposits and TAU protein neurofibrillary tangles (Murphy & LeVine, 2010). In recent years, it has been found that not only is the pathological accumulation of these proteins in brain tissues related to the onset and progression of the disease, but there is also an inflammatory response at the brain level induced through the signaling of microglial and astrocytic cells. It has been shown that Aβ is capable of causing the activation of microglia (Hansen et al., 2018) and the subsequent secretion of proinflammatory cytokines such as IL-1β and tumor necrosis factor α (TNFα), which promotes a conformational change in the astrocytes to a proinflammatory phenotype that signals the activation of transcription factors, induces the secretion of chemokines in brain tissue, and increases the migration of immune cells through a higher permeability of the blood–brain barrier (Leng & Edison, 2021); these inflammatory changes do not appear to occur only in the central nervous system. Differences in the concentration of various inflammatory markers have been reported in peripheral samples of patients with Alzheimer’s or mild cognitive impairment (MCI) compared to individuals with normal cognition (Sabbagh & Blennow, 2019).

Evidence has shown a relationship between systemic inflammatory processes and cognitive decline in normal aging and disease. IL-6 levels have been negatively associated with Mini-Mental State Examination (MMSE) scores in subjects with normal cognition (Wright et al., 2006), C-reactive protein levels have shown an inverse association with performance on the clock-drawing test (Ravaglia et al., 2003) and peripheral cytokine levels of IL-1β, IL-4R, IL-6, IL-8, IL-10, IL-12, and TNF-α have been associated with performance in cognitive domains of attention, cognitive speed, and short-term and long-term memory (Baune et al., 2008). An increase in the levels of inflammatory markers such as C-reactive protein and dysregulation in the secretion of proinflammatory cytokines like TNFα (12), IL-1β, IL-6, and multiple chemokines have also been associated with the decline in cognitive function observed in subjects with Alzheimer’s and MCI (13). Neuropathological changes along with the inflammatory process seem to be directly related to the progression of cognitive decline observed in AD and appear to be also a risk factor for its development (Kipinoinen et al., 2022). The evidence for an association between cognitive decline and systemic inflammation is ambiguous, so we aimed to estimate the association between blood levels of inflammatory markers and cognitive function in adults with Alzheimer’s disease or mild cognitive impairment.

## Methods

This review was conducted following the recommendations of the Cochrane Collaboration and following the PRISMA Statement.

### Eligibility Criteria

We included cohort, case–control, and cross-sectional studies whose population consisted of patients diagnosed with Alzheimer’s disease or mild cognitive impairment and met the following inclusion criteria: Studies reporting the levels of a cognitive function test and blood levels of inflammatory markers for a cohort with Alzheimer’s disease or mild cognitive impairment and their controls, studies reporting the correlation between blood levels of inflammatory markers and cognitive function levels in patients with Alzheimer’s disease or mild cognitive impairment. Exclusion criteria included studies in animal models or cell lines, populations with other neurodegenerative diseases, populations with risk for Alzheimer’s development, and samples with associated chronic diseases or without the diagnosis of interest. For studies that reported data from different population groups, data were taken only from the groups of interest.

### Information Sources

The literature search used Medical Subject Headings (MeSH), DeCS, and related text words. The search included all published literature in MEDLINE (OVID), WEB OF SCIENCE, SCOPUS, LILACS, and the Cochrane Central Register of Controlled Trials (CENTRAL) from their inception until April 2024. To ensure literature saturation, references of relevant articles identified through the search, thesis databases, Open Grey, and Google Scholar, among others, were scanned. There were no language or time limitations. The complete search strategy for each database is provided in Appendix 1.

### Study Selection

A double-masked review was performed. Two researchers independently reviewed the titles and abstracts of the articles within the systematic review to determine their potential usefulness. Eligibility criteria were applied during the full-text review of potentially eligible articles. Discrepancies were resolved by consensus between the two researchers. In cases without consensus, a third reviewer made the final decision.

### Data Collection

Data were collected by two researchers using a standardized extraction tool that included year and country of publication data, study design, group distribution, sample sizes, age breakdowns, gender distribution among groups, sample type, sample analysis method, main findings, cognitive function test scores, and concentrations of inflammatory markers for each group. For statistical analysis, means, standard deviations, standard errors, interquartile ranges, minimum and maximum ranges, and confidence intervals of inflammatory marker levels and cognitive function tests were extracted. All data were standardized to means and standard deviations, except for minimum and maximum ranges per Cochrane recommendations. In case of missing data, the corresponding authors were contacted. If no data were submitted, we excluded this study. All inflammatory marker levels were transformed to the same unit of measurement, pg/ml, except for C-reactive protein, which was converted to mg/dl. Data were cross-verified by reviewers.

### Risk of Bias and Methodological Quality Assessment

The Newcastle–Ottawa Scale (for cohort, case–control, and adapted to cross-sectional studies) was used to assess the methodological quality of the studies, following Cochrane Collaboration recommendations. The Newcastle–Ottawa Scale comprises eight items grouped into three dimensions with a scoring range of 0 to 9 points. Studies were classified as low quality (0–2 points), moderate quality (3 to 5 points), and high quality (6 to 9 points) (21).

### Data Analysis

Statistical analysis was performed using SPSS version 29.0.2.0 and Review Manager 5.3. The random-effects model calculated each outcome’s weighted mean differences and 95% confidence intervals. Heterogeneity was assessed using the I^2^ test, where values of < 50% and > 50% correspond to low and high levels of heterogeneity, respectively. Meta-regression analyses were conducted to compare mean differences in outcomes with Mini-Mental Test scores, age, and gender distribution. Publication Bias was undertaken to identify information or publication bias using a funnel plot. Sensitivity analysis was performed by extracting weighted studies and running estimated effects to find differences. Subgroup analysis was conducted using a sample type for each inflammatory marker to determine if blood fraction selection influenced the results.

## Results

### Study Selection

**Fig. 1:**
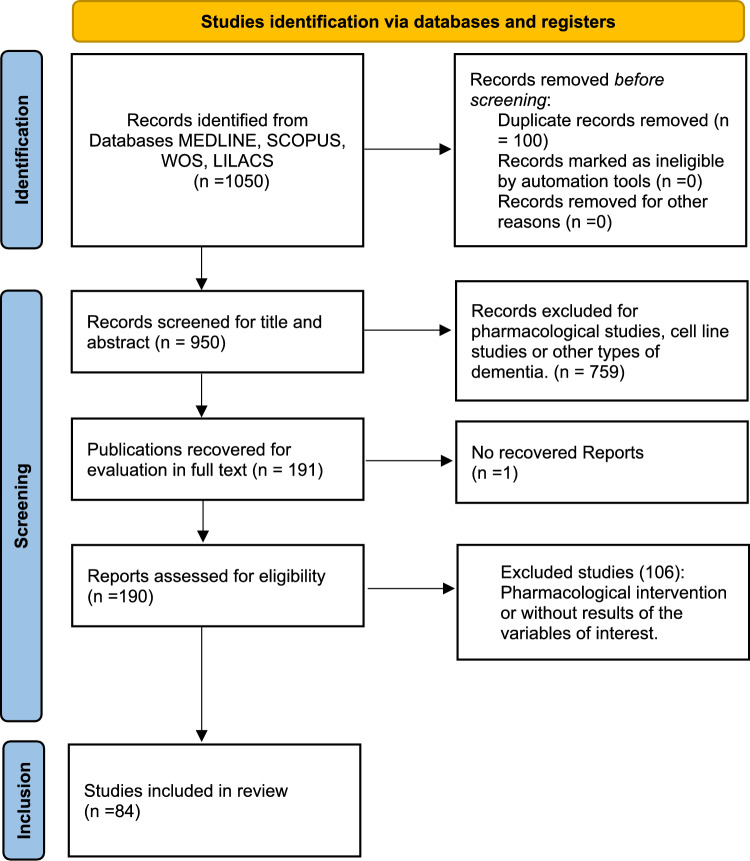
Flowchart

A total of 1050 studies were identified from the database search. After excluding duplicates, 950 studies were examined based on title and abstract, of which 759 were excluded for being pharmacological studies, studies on cell lines, or involving other types of dementia. One hundred ninety studies were selected for full-text review. Finally, it resulted in 84 studies in qualitative synthesis (Abe et al., 2020; Alsadany et al., 2013a; Angelopoulos et al., 2008; Björkqvist et al., 2012; Boccardi et al., 2019; Bonotis et al., 2008; Bossù et al., 2008; Cherbuin et al., 2019; Ciabattoni et al., 2007; Cisbani et al., 2020; Culjak et al., 2020; De Luigi et al., 2002; Delaby et al., 2015; Dubenko et al., 2021; Dursun et al., 2015; Fenoglio et al., 2004; Forlenza et al., 2009; Galimberti et al., 2006; Gezen-Ak et al., 2013; Gongora-Rivera et al., 2020; Gorska-Ciebiada et al., 2015; Gross et al., 2019; Gupta et al., 2017; Hazen et al., 2020; Hesse et al., 2016; Hochstrasser et al., 2012; Holmes et al., 2011; Huang et al., 2013; Julian et al., 2015; Karim et al., 2014; Kim et al., 2008, 2011, 2017; King et al., 2018, 2019; Koca et al., 2022; Kumar et al., 2023; Laske et al., 2008; Lawlor et al., 1996; Lee et al., 2018; Leung et al., 2013; Liang et al., 2021; Licastro et al., 2000; Lindberg et al., 2005; Llano et al., 2012; Mahdavi et al., 2021; Malaguarnera et al., 2006; Marksteiner et al., 2011; Mohd Hasni et al., 2017; Motta et al., 2007; Nie et al., 2022; O’Bryant et al., 2016; Paganelli et al., 2002; Park et al., 2021; Perea et al., 2018; Ramadan et al., 2022; Rasi Marzabadi et al., 2021; Reale et al., 2012; Richartz et al., 2005; Rota et al., 2006; Savaş et al., 2019; Scarabino et al., 2020; Schipke et al., 2019; Shateri et al., 2023; Shen et al., 2019; Soares et al., 2009; Startin et al., 2019; Sun et al., 2003, 2022; Tarkowski et al., 2001; Teunissen et al., 2003; Uslu et al., 2012; Vacinova et al., 2021; Villarreal et al., 2016; Wennberg et al., 2019; Westin et al., 2012; Wu et al., 2015; Xu et al., 2021; Yasutake et al., 2006; Zaciragic et al., 2007; Zhang et al., 2013; Zhao et al., 2012; Zuliani et al., 2007, 2008) and 75 in meta-analysis (Fig. 1).

### Characteristics of Excluded Studies

The excluded articles were not related to the intervention or outcomes of interest. Additionally, editorials, systematic or narrative reviews, drug intervention protocols, and animal studies were excluded.

### Characteristics of Included Studies

The 84 studies included in this review were published between 1996 and 2023, comprising 48 case–control studies, 25 cross-sectional studies, and 11 cohort studies. Among the 84 studies, 22 reported data on Alzheimer’s disease, mild cognitive impairment, and control groups (Table 1); 42 studies reported Alzheimer’s data and controls with normal cognition (Table 2) groups; 11 cohort studies reported data on cognitive impairment and normal cognition (Table 3) groups; 2 studies reported data from Alzheimer’s and mild cognitive impairment cohorts (Table 4) groups; and we included seven cross-sectional cohort studies without a control group, 6 of these studies reported results in subjects with Alzheimer’s disease and one in subjects with mild cognitive impairment (Table 4). All studies included both sexes participants and sample sizes ranged from 22 to 1602 participants, most of the studies had a higher number of women in Alzheimer’s or cognitive impairment patients’ groups.

**Table 1:** General characteristics of the studies with Alzheimer’s and normal cognition groups

Study	Type study	Type of sample	MEASURE METHOD	*n* total	AD			NC			MEASURE	
					*n*	Age	SEX (%F)	*n*	Age	SEX (%F)	Cognitive Function	Biomarker
Delaby et al., 2015* (FR)*	Case control	Serum	ELISA	55	31	70.84 ± 8.71	NR	24	66.63 ± 13.30	NR	MMSE	CRP, IL-3, IL-8, MCP-1, MIP-1β, CCL20, RANTES, TGF-β1
Huang et al., 2013* (TW)*	Cross sectional	Plasma	ELISA	47	28	83.00 ± 6.8	8,5	19	79.89 ± 7.0	10,6	MMSE	TNF-α, IL-6
Koca et al., 2022* (TR)*	Case control	Plasma	immuno-microarray	50	25	73.2 ± 5.0	22,0	25	65.8 ± 7.02	22,0	MMSE	IL-1α, IL-1β, IL-2, IL-4, IL-5, IL-6, IL-8, IL-10, IL-12, IL-13, GM-CSF, IFN- γ, MCP-1, CCL3, MIP-1β, RANTES, TNF-α
Mohd Hasni et al., 2017* (MY)*	Case control	Serum	ELISA	78	39	80.7 ± 6.41	28,2	39	72.1 ± 5.04	19,2	MMSE	IL-1β, IL-6, IL-12, IFN- γ, TNF-α, IL-10, CXCL-1, IL-8, CXCL10, MCP-1, MIP-1, IL-13
Richartz et al., 2005* (DE)*	Cross sectional	Plasma	ELISA	50	27	70	36,0	23	68	32,0	MMSE	IL-6, IL-12, IFN- γ, TNF-α
Savaş et al., 2019* (TR)*	Case control	Serum	ELISA	76	46	76.0 ± 9	40,8	30	72 ± 9	23,7	MMSE	TNF-α
Shateri et al., 2023* (IR)*	Case control	Plasma	ELISA	120	40	69.95 ± 5.43	19,2	40	74.32 ± 5.34	16,7	MMSE	IFN- γ, IL-1α, IL-1β, IL-2, IL-4, IL-6, IL-8, IL-12, TNF-α, IL-10, IP-10, MCP-1
Startin et al., 2019* (UK)*	Case control	Plasma	ELISA	54	27	59.33 ± 4.04	16,7	27	49.26 ± 10.40	20,4	MMSE	IL-1β, IL-10, IL-6, TNF-α
Sun et al., 2022* (CN)*	Cross sectional	Plasma	ELISA	74	30	75.27 ± 7.67	16,2	44	71.86 ± 7.76	29,7	MMSE	IL-1β, IL-6, IL-8, TNF-α
Uslu et al., 2012* (TR)*	Cross sectional	Plasma	ELISA	51	28	68.39 ± 6.74	35,3	23	66.17 ± 9.45	25,5	MMSE	IL-6, TNF-α
Vacinova et al., 2021* (CZ)*	Case control	Plasma	ELISA	163	85	75.7(73.2 76.8)	28,2	78	66(64.4 67.8)	33,1	RBANS	RANTES, IL-6, TNF-α, CRP
Rota et al., 2006* (IT)*	Case control	Serum	ELISA	55	30	71 ± 6.03	32,7	25	69 ± 8.60	18,2	MMSE	IL-12 p 40, IL- 12 p 70, IL-10, TGF-β1
Teunissen et al., 2003* (NL)*	Case control	Serum	ELISA	95	34	73 (53–95)	21,1	61	68 (55–86)	27,4	MMSE	IL-6, CRP, CC16
Zaciragic et al., 2007* (BA)*	Case control	Serum	ELISA	30	15	73.46 ± 2.57	NR	15	69.93 ± 2.57	NR	MMSE	CRP
Ciabattoni et al., 2007* (IT)*	Cross sectional	Plasma	ELISA	88	44	73 ± 8	28,4	44	75 ± 7	30,7	MMSE	TNF-α, CRP, IL-6
Zuliani et al., 2008* (IT)*	Case control	Plasma	ELISA	90	60	78,5 ± 7,6	43,3	30	74,5 ± 5,1	16,7	MMSE	TNF-α, IL-1β, IL-6, IFN- γ
Zuliani et al., 2007* (IT)*	Case control	Plasma	ELISA	102	60	78,5 ± 7,6	38,2	42	72,5 ± 5,8	18,6	MMSE	IL-1β, TNF-α, IL-6, IL-10
Licastro et al., 2000* (IT)*	Case control	Serum	ELISA	196	145	75 ± 1	15,8	51	78 ± 2	46,4	MMSE	IL-6, IL-1β
Angelopoulos et al., 2008* (GR)*	Cross sectional	Serum	ELISA	71	56	74 ± 8,6	NR	15	59,2 ± 9,1	NR	MMSE	IL-1β, TNF-α, IL-6, IL-10, IL-2
Bonotis et al., 2008* (GR)*	Cross sectional	Serum	ELISA	40	19	75,05 ± 4,9	27,5	21	71,23 ± 4,4	27,5	MMSE	IL-1α, IL-2, IL-6, IL-8, IL-10, TNF-α
Yasutake et al., 2006* (JP)*	Cross sectional	Serum	ELISA	93	60	77,9 ± 7,0	43,0	33	71,1 ± 5,8	26,9	MMSE	TNF-α, IL-1β
Lawlor et al., 1996* (IE)*	Cross sectional	Serum	ELISA	67	17	74,94 ± 5,34	17,9	50	68,47 ± 6,84	17,9	MMSE	CRP
Malaguarnera et al., 2006* (IT)*	Case control	Plasma	ELISA	70	40	73,2 ± 7,08	34,3	30	73,5 ± 3,24	21,4	MMSE	IL-18, TGF-β1
Motta et al., 2007* (IT)*	Case control	Plasma	ELISA	27	11	75,3 ± 5,2	14,8	20	70 ± 2,38	37,0	MMSE	IL-12, IL-16, IL-18, TGF-β1
Tarkowski et al., 2001* (SE)*	Case control	Serum	ELISA	50	20	63 ± 1	24,0	30	65 ± 2	NR	GBS scale	IFN- γ, IL-4, IL-8, IL-10, TGF-β1
Bossu et al., 2008* (IT)*	Cross sectional	Serum	ELISA	55	30	71,3 ± 1,6	25,5	25	68,5 ± 1,4	36,4	MMSE	IL-18
Alsadany et al., 2013* (EG)*	Cross sectional	Plasma	ELISA	50	25	7,22 ± 5,9	28,0	25	72,8 ± 4,1	26,0	MMSE	IL-8
Björkqvist et al., 2012* (SE)*	Cross sectional	Plasma	Citokine array	199	25	76 (56–87)	20,1	174	74 (62–99)	88,9	MMSE	RANTES, G-CSF, IL-1α, IL-3, IL-8, IL-11, CCL17, M-CSF, MIP-1, TNF-α
Fenoglio et al., 2004* (IT)*	Case control	Serum	ELISA	472	269	75 (51–101)	40,5	203	72 (46–96)	23,3	MMSE	MCP-1
Gongora et al., 2020* (MX)*	Case control	Serum	Luminex assay	58	29	75.34 ± 7.3	41,4	29	72.85 ± 6.59	63,8	MMSE	IL-1α, IL-3, IL-18, IL-16, IL-12, IFN- α2, CCL7, M-CSF, CXCL9, CCL27, TNF- B, CXCL12
Hesse et al., 2016* (DE)*	Case control	Serum	ELISA	60	36	68 (66–70)	41,7	24	70 (64–72)	20,0	MMSE	IL-1β, IL-8, TNF-α
Laske et al., 2008* (DE)*	Case control	Plasma	ELISA	60	30	70.5 ± 8.2	30,0	30	69.9 ± 11.1	16,7	MMSE	CXCL12
Llano et al., 2012* (US)*	Case control	Plasma	Citokine array	22	15	70.2 ± 7.4	13,6	7	65.0 ± 5.2	9,1	MMSE	IL-1β, IL-2, IL-6, IL-8, IL-10, IL-12, GM-CSF, IFN- γ, TNF-α
O’Bryant et al., 2016* (US)*	Case control	Serum/Plasma	Multiplex assay	144	79	76.1 ± 8.6	38,9	65	71.2 ± 9.2	24,3	MMSE	Eotaxin, CRP, IL-5, IL-6, IL-7, IL-10, IL-18, TNC, TNF-α
Perea et al., 2018* (ES)*	Case control	Plasma	ELISA	28	14	68 ± 4.18	32,1	14	64 ± 2.92	25,0	MMSE	CX3CL1
Reale et al., 2012* (IT)*	Case control	Plama	ELISA	77	38	73.8 ± 5.5	23,4	39	72.7 ± 4.8	27,3	MMSE	IL-18, MCP-1, RANTES
Schipke et al., 2019* (DE)*	Case control	Serum	ELISA	160	81	81.9 ± 7.8	17,5	79	64.5 ± 2.7	33,8	MMSE	TGF-β1, MCP-1, IL-18
Soares et al., 2009* (NL)*	Case control	Plasma	Luminex assay	41	19	81.0 ± 4.8	29,3	22	76.5 ± 7.5	34,1	MMSE	IL-3, RANTES, IL-8, TNF-α
Wu et al., 2015* (TW)*	Case control	Plasma	ELISA	81	41	73.1 ± 9.4	33,3	40	63.0 ± 5.6	33,3	MMSE	IL-6, IL-18, Fractalkine
Xu et al., 2021* (CA)*	Case control	Serum	Multiplex assay	480	212	73.44 ± 8.53	24,6	268	73.21 ± 8.71	30,8	MMSE	MCP-1
Zhang et al., 2013* (US)*	Case control	Plasma	ELISA	72	41	77.9 ± 7.7	33,3	31	75.4 ± 9.5	20,8	MMSE	MCP-1
Luigi et al., 2002* (SE)*	Case control	Plasma	ELISA	105	17	NR	NR	47	NR	NR	MMSE	TNF-α, IL-1β

Age is expressed mean and standard deviation; *NR* Not Reported, *MMSE* Minimental Test, GBS Scale Gottfries–Brane–Steen Geriatric Rating Scale, **CRP** C-reactive Protein, *IL* Interleukin, *IFN* Interferon, *TNF* Tumor Necrosis Factor, *TGF* Transforming Growth Factor, *GM-CSF* Granulocyte–Macrophage CSF, MCP-1 Monocyte Chemoattractant Protein 1, *CXCL* Chemokine (C–X–C motif) Ligand, MIP-1β Macrophage Inflammatory Protein, IP-10 Interferon Gamma-Induced Protein 10

**Table 2:** General characteristics of the studies with Alzheimer’s, mild cognitive impairment, and normal cognition groups

Study (Setting)	Type study	Type of sample	MEASURE METHOD	*n* total	AD			MCI			NC			MEASURE	
					*n*	Age	SEX (%F)	*n*	Age	SEX (%F)	*n*	Age	SEX (%F)	Cognitive Function	Biomarker
Boccardi et al., 2019* (IT)*	Case control	Plasma	Multiplex assay	289	129	81,0 ± 6,2	59,8	73	77,5 ± 6,3	64,3	87	75,9 ± 9.0	46,4	MMSE	IFN- α2, IL-1α, MCP-1
Cisbani et al., 2020* (CA)*	Case control	Serum	Cytokines array	32	11	65.7 ± 3.5	54,5	10	70.91 ± 1.6	50,0	11	70.0 ± 2.1	54,5	MMSE, MoCA, FAS Test	IL-1α, IL-1β, IL-5, IL-6, IL-10, IL-17, IFN- γ, TNF-α
Hochstrasser et al., 2012* (AT)*	Cross sectional	Plasma	ELISA	103	34	79 ± 1.1	82,4	43	72 ± 1.2	62,8	26	72 ± 1.2	46,2	MMSE	IL-1β, IL-11
Kim et al., 2017* (KR)*	Case control	Serum	ELISA	92	35	77.66 ± 8.11	82,9	29	75.03 ± 7.46	62,1	28	72.00 ± 7.28	57,1	MMSE, CDR	TNF-α, IL-6
King et al., 2018* (UK)*	Cross sectional	Plasma	ELISA	61	20	75.9 ± 6.7	25,0	21	78.5 ± 6.4	66,7	20	75.9 ± 7.3	20,0	MMSE, ACE	CRP, IFN- α2, IL-10, IL-1β, IL-2, IL-4, IL-6, IL-8, TNF-α
Leung et al., 2013* (SE)*	Cohort	Plasma	ELISA	341	112	76.2 ± 6.09	66,7	112	73.9 ± 5.63	49,2	117	72.3 ± 6.72	53,6	MMSE	IL-1β, IL-1α, IL-2, IL-4, IL-5, IL-6, IL-7, IL-8, IL-9, IL-10, IL-12, IL-13, IL-15, IL-17, Eotaxin, FGF, G-CSF, GM-CSF, IFN- γ, IP-10, MCP-1, TNF-α
Liang et al., 2021* (TW)*	Cohort	Plasma	ELISA	91	28	78.3 ± 8.8	75,0	51	75.6 ± 8.6	78,8	12	66.3 ± 5.9	75,0	MMSE, CDR	IL-2, IFN- γ, TNF-α, IL-4, IL-5, IL-6, IL-10, IL-13, IL-1β, IL-17A, IL-23, IL-25, IL-31, IL-8, IP-10, MCP-1, CCL3, MIP-1β, RANTES, Eotaxin, IL-7, IL-9, G-CSF, GM-CSF
Park et al., 2021* (KR)*	Cross sectional	Plasma	ELISA	79	26	75.54 ± 6.17	76,5	28	75.60 ± 6.15	50,0	25	75.56 ± 6.29	44,0	MMSE	IL-1β, TGF-β1, CRP
Scarabino et al., 2020* (IT)*	Case control	Plasma	ELISA	472	254	78.5 ± 8.2	70,9	56	76.1 ± 6.0	60,7	162	70.0 ± 8.9	61,1	MMSE	IL-1β, IL-18
Villarreal et al., 2016* (PA)*	Cross sectional	Serum	ELISA	135	28	81.9 ± 9.2	78,6	30	81.2 ± 7.8	66,7	77	76.5 ± 6.7	64,9	MMSE	CRP, Eotaxin, TNF-α, TNC, IL-5, IL-6, IL-7, IL-10, IL-18, IL-1β, CCL3
Dursun et al., 2015* (TR)*	Case control	Serum	ELISA	157	EO:21; LO:53	EO: 60,86 ± 4,76; LO:74,02 ± 3,9	NR	30	74,4 ± 2,9	NR	EO:21;LO:32	EO: 57,95 ± 6,39; LO: 72,1 ± 3,4	NR	MMSE	IL-1α, IL-1β, IL-6
Lindberg et al., 2005* (SE)*	Case control	Serum	ELISA	43	12	73,6 ± 2,75	66,7	20	60,9 ± 1,89	55,0	11	58,1 ± 2,23	45,5	MMSE	IL-18
Forlenza et al., 2009* (BR)*	Case control	Serum	ELISA	163	58	76,3 ± 6,5	82,8	74	70,7 ± 10,3	74,3	31	69,9 ± 6,7	64,5	MMSE, CAM cog, VFT	IL-1β
Galimberti et al., 2006* (IT)*	Case control	Serum	ELISA	163	58	75,0 ± 0,71	74,5	74	73,3 ± 0,9	60,4	31	nr	70,8	MMSE	MCP-1
Gupta et al., 2017* (AU)*	Cohort	Serum	ELISA	711	92	77.01 ± 7.43	64,2	65	74.84 ± 7.54	55,4	554	69.79 ± 6.51	59,6	MMSE	IL-18
Kim et al., 2008* (KR)*	Cross sectional	Plasma	ELISA	159	51	78.2 ± 6.1	82,4	51	74.6 ± 7.0	80,4	57	70.5 ± 3.8	70,2	MMSE, GDS	Fractalkine
Kim et al., 2011* (KR)*	Case control	Plasma	ELISA	59	18	75.9 ± 6.0	50,0	20	76.1 ± 2.8	55,0	21	75.5 ± 1.3	52,4	MMSE, GDS	IL-8, TNF-α, IL-10, MCP-1
Lee et al., 2018* (TW)*	Cohort	Plasma	ELISA	496	310	80.1 ± 7.2	57,7	66	75.4 ± 8.2	47,0	120	74.9 ± 7.8	45,8	MMSE	MCP-1
Marksteiner et al., 2011* (AT)*	Case control	Plasma	Protein Array	159	96	77.0 ± 0.8	NR	44	73.5 ± 1.2	NR	19	72.1 ± 1.3	NR	MMSE	G-CSF, IL-1α, IL-3, IL-8, IL-11, CCL7, M-CSF, CCL15, CCL18, RANTES, TNF-α
Westin et al., 2012* (SE)*	Cohort	Plasma	ELISA	129	47	74 ± 6	21,4	52	64 ± 9	46,2	30	72 ± 8	56,7	MMSE	CCL2, CCL11, CCL13, CCL26
Dubenko et al., 2021 (UA)	Case control	Serum	ELISA	75	15	67.9 ± 0.8	66,7	30	65.6 ± 0.8	40,0	30	65.7 ± 0.9	NR	MMSE	IL-17, IL-23
Gezen et al., 2013 (TR)	Case control	Serum	ELISA	156	EO:22; LO:54	EO: 61,1 ± 4,8; LO:74,22 ± 3,73	NR	30	74,4 ± 2,9	NR	EO:18;LO:32	EO: 58,6 ± 7,0; LO: 72,1 ± 3,4	NR	MMSE	TNF-α, IL-10

Age is expressed mean and standard deviation; *NR* Not Reported, *MMSE* Mini-Mental Test, *VFT* Verbal Fluency Test, *ACE* Addenbrooke’s Cognitive Examination, *FAS* F-A-S Verbal Fluency Test, *CDR* Clinical Dementia Rating, ADAS-Cog Test ADAS-COG, *MoCA* Montreal Cognitive Test, *GBS* Gottfries–Brane–Steen Geriatric Rating Scale, *CRP* C Reactive Protein, *IL* Interleukin, *IFN* Interferon, *TNF* Tumor Necrosis Factor, *TGF* Transforming Growth Factor, *GM-CSF* Granulocyte–Macrophage CSF, *MCP-1* Monocyte Chemoattractant Protein 1, *CXCL* Chemokine (C–X–C motif) Ligand, *MIP-1β* Macrophage Inflammatory Protein, *IP-10* Interferon Gamma-Induced Protein 10

**Table 3:** General characteristics of the studies with Mild cognitive impairment and normal cognition groups

Study (Setting)	Type study	Type of sample	MEASURE METHOD	*n* total	MCI			NC			MEASURE	
					*n*	Age	SEX (%F)	*n*	Age	SEX (%F)	Cognitive Function	Biomarker
Abe et al., 2020* (JP)*	Cohort	Plasma	Luminex assay	343	296	74.73 ± 7.14	35,1	47	75.36 ± 5.89	48,9	MMSE, ADAS cog	CNTF, CRP, IL-13, IL-16, IL-18, IL-3, IL-6R, IL-8, CXCL10, TNF-α
Cherbuin et al., 2019* (AU)*	Case control	Plasma	ELISA	380	81	75.48 ± 1.54	44,5	299	75.31 ± 1.37	NR	MMSE	IL-1β, IL-4, IL-6, IL-8, IL-10
Gross et al., 2019* (US)*	Cohort	Plasma	ELISA	191	23	70.4 ± 6.9	39,1	168	64.2 ± 8.7	65,5	General cognitive factor	IL-6
Wennberg et al., 2019* (US)*	Cohort	Plasma	Simoa	1602	186	79.7 (73.1, 84.7)	40,9	1416	71.9 (63.6, 78.4)	47,2	Global score	IL-6, IL-10, TNF-α
Kumar et al., 2023* (IN)*	Cross sectional	Plasma	ELISA	105	28	75.14 ± 1.24	28,6	77	73.18 ± 1.43	28,6	MMSE, MoCA	IL-6, IL-1β, TNF-α
Zhao et al., 2012* (CN)*	Case control	Serum	ELISA	300	150	70.67 ± 4.27	47,3	150	69.85 ± 5.21	41,3	MMSE, MoCA	IL-6, IFN- γ, TNF-α, IL-10
Nie et al., 2022* (CN)*	Case control	Plasma	Luminex assay	46	23	73.0 ± 4.5	56,5	23	72.5 ± 0.8	60,9	MoCA	IL-6, CXCL11, CCL13, CXCL16, CCL15, TNF-α, CCL1, CXCL8, CCL7, MIP-2, IL-1β, IFN- γ, CXCL5, CXCL6, CCL25, CCL2, CCL17
King et al., 2019 (UK)	Cross sectional	Plasma	Citokine assay	41	21	78,5 ± 6,4	66,7	20	75,9 ± 7,3	20,0	MMSE	CRP, IFN- γ, IL-10, IL-1β, IL-2, IL-4, IL-6, IL-8, TNF-α
Shen et al., 2019 (SG)	Case control	Plasma	Luminex assay	114	57	68.77 ± 5.47	31,6	57	67.77 ± 5.16	31,6	MMSE	TNF-α, IP-10, CXCL13, hsCRP, IL-8, MCP-1, IL-4
Ramadan et al., 2022 (EG)	Case control	Serum	ELISA	90	45	70.84 ± 6.98	42,2	45	71.51 ± 6.0	48,9	MMSE	CRP, IL-10
Gorska-Ciebiada et al., 2015 (PL)	Cross sectional	Serum	ELISA	194	62	74.7 ± 3.9	51,6	132	72.5 ± 4.9	62,1	MoCA	CRP, IL-6, TNF-α

Age is expressed mean and standard deviation; *NR* Not Reported; *MMSE* Mini-mental test, *ADAS cog* Test ADAS-COG, *FAS* F-A-S verbal fluency test, *MoCA* Montreal Cognitive Test, *GBS* Gottfries–Brane–Steen Geriatric Rating Scale, *CRP* C Reactive Protein, *IL* Interleukin, *IFN* Interferon, *TNF* Tumor Necrosis Factor, *TGF* Transforming Growth Factor, *GM-CSF* Granulocyte–macrophage CSF, *MCP-1* Monocyte Chemoattractant Protein 1, *CXCL* Chemokine (C–X–C motif) Ligand, *MIP-1β* Macrophage Inflammatory Protein, *IP-10* Interferon Gamma-Induced Protein 10

**Table 4:** General characteristics of cross-sectional studies

Study (Setting)	Type study	Type of sample	MEASURE METHOD	N SAMPLE	Age (years) Mean ± SD		MEASURE	
						SEX (%F)	Cognitive Function	Biomarker
Paganelli et al., 2002* (IT)*	Cross sectional	Serum	ELISA	AD Severe = 11	74,7 ± 2,2	45,4	MMSE	TNF-α, IL-1β
				AD Mild = 25	74,1 ± 1,8	36		
Sun et al., 2003* (SE)*	Cross sectional	Plasma	ELISA	AD = 141	75 (52–85)	53,1	MMSE	IL-6, MCP-1
Rasi Marzabadi et al., 2021* (IR)*	Cross sectional	Serum	ELISA	Mild AD = 30	74.90 ± 5.66	86,6	MMSE	TNF-α, IL-6, IL-1β
				AD mild-moderate = 31	75.90 ± 6.56	77,4		
				AD moderate-Severe = 19	77.94 ± 6.32	68,4		
Mahdavi et al., 2021* (IR)*	Cross sectional	Serum	ELISA	AD = 39	69.95 ± 5.43	56,4	MMSE, CDR, ADAS cog	CRP, TNF-α, IFN- γ, IL-1α, IL-1β, IL-2, IL-4, IL-6, IL-8, IL-10, IL-12, IP-10, MCP-1
Karim et al., 2014* (UK)*	Cohort	Plasma	ELISA	MCI = 70	69,8 (68–72)	40	MMSE, FAS	IL-6, CRP
Julian et al., 2015* (FR)*	Cross sectional	Plasma	Luminex assay	AD = 109	79.44 ± 6.82	72,4	MMSE, ADAS cog	IL-1β, IL-6, TNF-α, RANTES
Holmes et al., 2011* (UK)*	Cohort	Plasma	ELISA	AD = 275	82.7 ± 7.4	61,8	ADAS cog	CRP, TNF-α, IL-6
Hazen et al., 2020* (NO)*	Cohort	Serum	ELISA	MCI (*n* = 88)	71.3 ± 10.3	47,7	MMSE	CCL2, IL-18, IL-6, MIP-1β, IL-1α, TNF-α, CRP
				AD (*n* = 154)	74.9 ± 7.4	85,7		
Culjak et al., 2020 (HR)	Cross sectional	Serum	ELISA	MCI (*n* = 250)	71.95 ± 4.88	62	MMSE	TNF-α, IL-1α, IL-10
				AD (*n* = 395)	78.83 ± 2.21	64		

Age is expressed mean and standard deviation; *NR* Not Reported, *MMSE* Mini-Mental Test, *ADAS cog* Test ADAS-COG, *MoCA* Montreal cognitive test, *GBS* Gottfries–Brane–Steen Geriatric Rating Scale, *CRP* C Reactive Protein, *IL* Interleukin, *IFN* Interferon, *TNF* Tumor Necrosis Factor, *TGF* Transforming Growth Factor, *GM-CSF* Granulocyte–Macrophage CSF, *MCP-1* Monocyte Chemoattractant Protein 1, *CXCL* Chemokine (C–X–C motif) Ligand, *MIP-1β* Macrophage Inflammatory Protein, *IP-10* Interferon Gamma-Induced Protein 10

Inflammatory markers across the 84 included studies were assessed in 47 studies in plasma samples, 36 studies analyzed serum samples, and one study reported marker levels in both plasma and serum samples. Regarding analysis methods, 68 studies used specific ELISA kits for each marker, six utilized protein microarrays, cytokines, or immunological assays, nine employed Luminex or Multiplex assays, and one study used Simoa (Tables 1, 2, 3, 4).

### Risk of Bias Assessment

The presentation of methodological evaluation results using the Newcastle–Ottawa Scale (NOS) is illustrated in Tables S1, S2, and S3. Overall, the studies showed good/high methodological quality. The 48 case–control studies had an average score of 6 on the NOS, with higher risks observed in control definition and case representativeness. Cross-sectional studies had an average score of 7, with higher risks in sample selection and definition of excluded subjects. Cohort studies had an average score of 6, with higher risks noted in areas related to follow-up time.

### Cognitive Function Assessment

Cognitive function was reported in the studies through multiple tests: Mini-Mental State Examination (MMSE), ADAS-COG test, F-A-S verbal fluency test (FAS), Clinical Dementia Rating (CDR), Addenbrooke’s Cognitive Examination (ACE), Buschke’s Memory Impairment Screen (MIS), Rivermead Behavioral Memory Test (RBMT), Montreal Cognitive Assessment (MOCA), Modified Boston Naming Test (MBNT), and general cognitive factor scores designed by the authors (Tables 1, 2, 3, 4).

The Mini-Mental State Examination (MMSE) was the most used test across studies, with 78 studies reporting scores for different groups. As expected, individuals with Alzheimer’s disease and Mild Cognitive Impairment (MCI) had lower scores compared to controls with normal cognition. Individuals with Alzheimer’s disease showed an average difference of − 10 points (*p* < 0.00001) compared to individuals with normal cognition. Individuals with MCI showed an approximate difference of − 3 points (*p* < 0.00001) compared to controls. Between individuals with Alzheimer’s disease and those with MCI, there was an approximate difference of − 8 points (*p* < 0.00001) (Table S4).

The Montreal Cognitive Assessment (MOCA) was the second most reported test. Five studies reported cognitive function assessments using this test, mainly in MCI subjects, with an average difference of approximately − 5 points (*p* 0.0008) in mild cognitive impairment cohorts compared to controls (Fig S1).

The other tests that could not be grouped were reported in 19 studies. Six studies reported ADAS-COG measures, most of them cross sectional (Abe et al., 2020; Holmes et al., 2011; Julian et al., 2015; Karim et al., 2014; Leung et al., 2013; Mahdavi et al., 2021); verbal fluency test (FAS) was reported in five studies (Alsadany et al., 2013; Cisbani et al., 2020; Forlenza et al., 2009; Karim et al., 2014; Liang et al., 2021); Four studies reported GDS score values (Kim et al., 2008, 2011, 2017; Tarkowski et al., 2001); two studies reported scores designed by the authors (Gross et al., 2019; Wennberg et al., 2019); and the remaining tests were reported in one study (Tables 1, 2, 3, 4).

### Levels of Inflammatory Markers

Sixty-five inflammatory markers were reported in the studies, comprising 34 chemokines, 24 interleukins, two tumor necrosis factors, two interferons, two colony-stimulating factors, and one acute phase protein. Mean and standard deviation values reported for each biomarker across studies were grouped for meta-analysis, involving 73 studies for statistical analysis (Tables 1, 2, 3, 4).

Significantly higher levels of IL-1β were detected in adults with Alzheimer’s disease compared to individuals with Mild Cognitive Impairment and controls with normal cognition (Fig S2). TNF-α and MCP-1 were found in elevated concentrations in patients with AD and MCI compared to controls with normal cognition (Fig S3a, b and S4a, b). Still, no significant difference was found between the AD and MCI groups. IL-6 was detected with significantly higher concentrations in Alzheimer’s patients compared to those with normal cognition (Fig S5). The only markers found to decrease were IL-10, detected at lower levels in subjects with AD compared to those with normal cognition (Fig S6), and IL-8, detected at low concentration levels in all group comparisons (Fig S7a–c) (Table 5).

**Table 5:** Inflammatory markers with significant difference between AD or MCI and normal cognition

Biomarker	# studies included	n	Main Effect				Heterogeneity			Regulation described
Comparison AD/NC		**AD/NC**	**MD**	**95% CI**	**z**	***p***	**Chi**^**2**^	***p***	**I**^**2**^	
IL-1β	20	1091/789	0,46	0,35, 0,58	7,82	< 0.00001	20032,32	< 0.00001	100	**↑**
IL-6	22	999/820	3,41	3,05, 3,78	18,31	< 0.00001	11,319,6	< 0.00001	100	**↑**
IL-8	15	578/523	− 1,46	− 1,85, − 1,08	7,43	< 0.00001	1928,63	< 0.00001	99	**↓**
IL-10	14	479/433	− 3.20	− 4.21, − 2.20	6,24	< 0.00001	1929,76	< 0.00001	99	**↓**
MCP-1	11	855/787	26,20	14,57, 37,82	4,42	< 0.00001	452,37	< 0.00001	98	**↑**
TNF-α	27	1215/1035	6,68	5,97, 739	18,36	< 0.00001	41,818,93	< 0.00001	100	**↑**
Comparison MCI/NC		**MCI/NC**								
IL-8	6	570/475	− 1,40	− 2,12, − 0,68	3,61	0.0001	2172,33	< 0.00001	100	**↓**
TNF-α	15	1043/2122	9,26	7,45,11,07	10,04	< 0.00001	17,762,07	< 0.00001	100	**↑**
MCP-1	5	275/208	35,07	1,58, 68,56	2,05	0,04	358,13	< 0.00001	100	**↑**
Comparison AD/MCI		**AD/MCI**								
IL-1β	9	512/343	0,21	0,11, 0,31	4,19	< 0.00001	128,97	< 0.00001	94	**↑**
IL-8	4	162/136	− 1,51	− 2,55, − 0,47	285	285	132,69	< 0.00001	98	**↓**

*AD* Alzheimer’s Disease, *MCI* Mild Cognitive Impairment, *MD* Mean Difference, IL Interleukin, *TNF* Tumoral Necrosis Factor, *MCP- 1* Monocyte Chemoattractant Protein 1

**Fig. 2:**
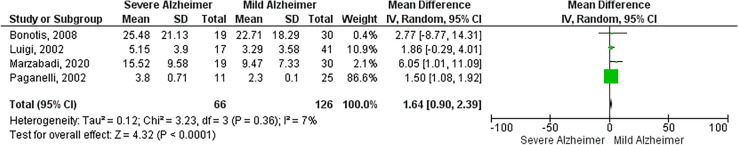
Analysis of levels of TNF-α in subjects with severe and moderate Alzheimer’s. Meta-analysis plot summarizing the effect sizes (with 95% confidence intervals) of levels of TNF-α on mild and severe Alzheimer’s subjects. Each horizontal line represents an individual study, with the square indicating the effect size and the line representing the confidence interval. The square size reflects the study’s weight in the meta-analysis. The diamond at the bottom represents the pooled effect size and its confidence interval

One of the biomarkers that showed significant differences between Alzheimer’s disease, mild cognitive impairment, and control groups was TNF-α. Four of the included studies in the review reported measures of this marker across different stages of Alzheimer’s severity. A statistical analysis was conducted with these studies to assess if there was a difference in TNF-α concentrations between patients with severe AD and mild-moderate AD, finding that individuals with severe AD had significantly higher levels of TNF-α compared to those with mild-moderate AD (Fig. 2).

### Sensitivity and Subgroups Analysis

Sensitivity analyses showed that in the IL-10 analysis, the outlier from the Mohd Hasni et al., 2017 study was the only one capable of significantly changing the combined result, resulting in a loss of significance (MD = − 0.0 (− 0,20, 0,19), I2 = 64%, 13 studies). According to the sensitivity analysis, the behavior of the rest of the markers was not significantly influenced by any study.

We found significant heterogeneity in all comparison groups despite all concentrations of inflammatory markers being measured in the same unit (pg/ml). Analyses were repeated in subgroups based on the type of sample used for marker measurement. For TNF-α, heterogeneity was slightly reduced in serum samples when the AD/NC groups were compared. Still, the significance of the association of high TNF-α levels in AD was lost (I2 = 86%, N = 409/343, in ten studies). The association of high concentrations of TNF-α, IL-1β, IL-8, IL-6, and MCP-1 in plasma remained, and the heterogeneity of the analyses did not change compared to previous analyses. However, the levels of IL-6 (I2 = 100%, N = 375/370, in nine studies) and MCP-1 (I2 = 100%, N = 690/644, in six studies) in serum lost significance while maintaining a high level of heterogeneity. The concentrations of IL-8 in serum in AD showed a change in directionality in the association; however, this change was influenced by one study (Tarkowski et al., 2001) (I2 = 99%, N = 145/138, in five studies).

TNF-α in plasma for patients with MCI maintained significance with a high level of heterogeneity (I2 = 100%, N = 669/1635, in 8 studies), while the analysis in serum showed a loss of significance with a high level of heterogeneity (I2 = 97%, N = 287/398, in five studies). Subgroup analysis could not be performed for IL-8 in serum due to insufficient articles available for this subgroup analysis.

Meta-regression analyses were conducted to assess the association between mean differences and MMSE scores, as well as the age and gender distribution of subjects with AD or MCI. The mean differences between subjects with AD and normal cognition controls in TNF-α concentrations (coefficient = − 0.341, 95% confidence interval: − 0.875, − 0.036; *p* = 0.037) and IL-1β (coefficient = − 0.456, 95% confidence interval: − 0.875, − 0.036) were correlated with MMSE scores. IL-1β (coefficient = 0.269, 95% confidence interval: 0.125–0.413) was also associated with age.

### Risk of Selective Reporting and Publication Bias

Significant risks of publication bias were not found in the evaluation of funnel plots; articles are distributed relatively evenly on the graphs, and most studies reported results of multiple markers and documented at least one negative result or lack of correlation between markers and cognitive function assessment, suggesting a low level of selection bias.

### Other Inflammatory Markers

Among the markers that could not be included in the meta-analysis, four studies reported significant differences between individuals with AD and controls, where CCL27 (Gongora-Rivera et al., 2020), CXCL12 (Gongora-Rivera et al., 2020), and CXCL10 (Mohd Hasni et al., 2017) were increased. At the same time, CCL3 (Koca et al., 2022), CCL15 (Marksteiner et al., 2011), CCL18 (Marksteiner et al., 2011), and IL-13 (Mohd Hasni et al., 2017) decreased in AD patients compared to controls.

Two studies reported significantly different concentrations of markers in individuals with MCI: CXCL11, CCL13, and CCL15 (Nie et al., 2022) were increased, and CXCL16 (Nie et al., 2022) and IL-16 (Abe et al., 2020) were decreased in individuals with mild cognitive impairment compared to controls.

### Correlation of Cross-Sectional Studies

Two of the cross-sectional studies included in this review reported that TNF-α has a positive correlation with ADAS-COG and MMSE (Holmes et al., 2011; Rasi Marzabadi et al., 2021), One study reported negative correlations between MMSE and TNF-α/IL-10 ratio (Mahdavi et al., 2021), and four studies reported no correlation between inflammatory markers and MMSE score (Julian et al., 2015; Karim et al., 2014; Paganelli et al., 2002; Sun et al., 2003).

## Discussion

### Summary of the Main Findings

This meta-analysis reveals higher TNF-α, MCP-1, IL-1β, and IL-6 levels in individuals with AD compared to controls with normal cognition. TNF-α and MCP-1 were also higher in MCI subjects compared to controls. The strongest associations with cognitive decline in AD and MCI were detected for IL-1β and TNF-α levels. IL-8 was lower in AD and MCI than in controls and was the only marker that remained consistently significant across all three comparison groups. At the same time, IL-10 was decreased in AD patients but not in those with MCI, with the association of IL-10 with AD being weaker. No significant differences were found in the concentrations of other studied markers, even after subgroup analysis.

### Contrast with the Literature

High levels of TNF-α and IL-1β have been previously reported in cerebrospinal fluid and peripheral samples of AD patients (Swardfager et al., 2010). IL-1β is considered a primary driver of the inflammation process, and elevated levels have been reported previously in the brains and peripheral samples of AD patients (Cacabelos et al., 1994). Elevated TNF-α levels have been linked to the progression from MCI to dementia (Fu et al., 2022) and have been implicated in adverse effects on functional connectivity in brain regions involved in spatial processing, memory, and language (Contreras et al., 2020). Evidence suggests that these cytokines are released early in AD and contribute to neurological decline by promoting enhanced Aβ plaque formation, tau phosphorylation, and more significant cholinergic system (Harry & Kraft, 2008).

IL-1β, IL-6, and TNF-α can stimulate their production mutually and act together in processes related to memory processing and learning (Bourgognon & Cavanagh, 2020). In Alzheimer’s disease, it appears that levels of these interleukins increase as the disease progresses and are associated with a higher risk of developing dementia in the early stages (Zhao et al., 2024). MCP-1 is a chemokine expressed by microglia and macrophages with proinflammatory characteristics. Its function includes regulating the migration of monocytes and macrophages, and it has been implicated in Aβ clearance. Studies have demonstrated that high levels of MCP-1 are associated with faster cognitive decline in patients with Alzheimer’s disease (Lee et al., 2018).

IL-10, a cytokine with anti-inflammatory characteristics, is released by both microglia and astrocytes in response to increased proinflammatory cytokines to maintain immune system homeostasis (Porro et al., 2020). We report low levels of IL-10 in AD subjects compared to control subjects with normal cognition, and evidence shows contradictory results in the levels of this marker. Elevated levels of IL-10 have been previously reported in Alzheimer’s patients. They are associated with poorer performance in cognitive tests, possibly linked to the exacerbation of Aβ pathology due to altered regulation in Aβ response and clearance (Sanchez-Molina et al., 2022). Nonetheless, low levels of IL-10 have also been reported previously. In animal models, deletion of the gene encoding IL-10 showed a worsening inflammatory process, leading to an increased inflammatory response (Weston et al., 2021), suggesting that the low levels reported in this meta-analysis could be related to weak expression of anti-inflammatory cytokines, including IL-10, which may expose subjects to a higher susceptibility to developing the disease.

The evidence supporting the correlation between IL-8 and cognitive decline is inconsistent. Some studies showed significantly higher levels of IL-8 in patients with AD and MCI. Others reported lower levels of IL-8 compared to subjects with normal cognition. Most studies, however, fail to demonstrate a correlation with cognitive assessment tests in this population (Alsadany et al., 2013; Doroszkiewicz et al., 2021). Studies, such as that by Luo et al., 2022, indicate that IL-8 levels are associated with better fluid intelligence but a smaller hippocampal volume, showing conflicting results regarding the levels of this cytokine and its association with cognitive decline (Luo et al., 2022). IL-8 is a proinflammatory cytokine involved in neutrophil chemotaxis and angiogenesis (Capogna et al., 2023). In this study, we observed lower levels of IL-8 in subjects with AD and MCI compared to controls, which may indicate a protective mechanism controlling neutrophil migration to reduce disease progression (Bawa et al., 2020). Additionally, IL-8 is considered a potent angiogenic factor, and pathological angiogenesis has been documented in Alzheimer’s disease (Vagnucci & Li, 2003), suggesting that low levels of this cytokine could play a role in regulating this process.

The relationship between peripheral inflammation observed in AD and the neuroinflammatory process needs to be clarified. Inflammation triggered by infections chronic or systemic diseases induces the production of inflammatory mediators such as IL-1β, TNF-α, and IL-6, and these proinflammatory mediators could signal the central nervous system through neural and humoral pathways, including crossing the blood–brain barrier, signaling via circumventricular organs, binding to endothelial receptors, and stimulating neural immune cells regulated by the vagus nerve (Xie et al., 2022). Studies in animal models have demonstrated that systemic infections can lead to cognitive impairment in individuals with neurological diseases and even in healthy adults (Perry et al., 2003). All the above indicate that peripheral inflammation may play an active role in disease progression and cognitive decline.

### Conceptual Integration and Temporal Progression

The findings of this study outline a possible chronology in which certain peripheral inflammatory markers are differentially associated with the stages of cognitive impairment. In early stages such as MCI, a moderate increase in cytokines such as TNF-α and MCP-1 is observed, possibly reflecting compensatory immune activation. As MCI progresses, IL-1β and IL-6 levels increase more pronouncedly, indicating an intensification of the systemic proinflammatory state. In parallel, the reduction of IL-10 and IL-8 could reflect a loss of anti-inflammatory control and an alteration of the neuroimmune axis. This progression suggests that peripheral inflammation not only accompanies cognitive impairment but could play an active role in its transition to more severe forms.

### Strengths and Limitations

This meta-analysis identifies a significant association between peripheral levels of six cytokines and cognitive decline in patients with AD and MCI. However, we encountered several limitations. Many studies reported substantial variations in the levels of each inflammatory marker, contributing to the observed high heterogeneity across all comparisons. Furthermore, some studies were excluded from the statistical analysis due to insufficient data that could be transformed into means and standard deviations. Despite investigating potential sources of heterogeneity, such as age and sex, subgroup analyses for a method of sample analysis were not possible due to inadequate data on sample analysis procedures for comparison.

Although the Mini-Mental State Examination (MMSE) is common for assessing cognitive impairment, it is vital to recognize its limitations as a general tool. The MMSE may lack sensitivity for detecting mild or specific deficits, particularly in executive or visuospatial domains. This limitation becomes more relevant considering that the included studies employed different cognitive assessment tools, such as the Montreal Cognitive Assessment (MoCA) or the Alzheimer’s Disease Assessment Scale-Cognitive Subscale (ADAS-Cog), each with varying approaches and sensitivities. To address this heterogeneity, the synthesis of results considered the type of tool used, and when possible, a stratified analysis was performed or differences discussed in qualitative terms. This variability in the instruments may have contributed to the heterogeneity observed in the findings. It should be considered when interpreting the magnitude and consistency of the reported effects.

Regarding the methodological quality, although the Newcastle–Ottawa Scale (NOS) was used to assess it, it is essential to consider how these differences in quality may have influenced the findings. In particular, cohort studies achieved higher NOS scores due to their longitudinal design, clearly defined selection criteria, and adequate follow-up periods. These characteristics contributed to stronger associations with greater temporal validity. In contrast, cross-sectional studies, which often received lower scores, were limited by their inability to establish temporality and were more susceptible to selection and reporting biases. These methodological disparities could explain the heterogeneity observed in the results, especially concerning variables related to exposure–response relationships.

Although high levels of heterogeneity were recognized, it is vital to consider other sources beyond demographic factors. Variations in laboratory standards by region, unadjusted comorbidities, and differences in assay methods (such as ELISA vs. Luminex) may have influenced the reported levels of biomarkers. These differences may affect reproducibility and limit the clinical applicability of the findings. Future research should standardize measurement methods and better control for these factors to improve the external validity of the results.

### Importance for Public Health

The growing body of evidence linking peripheral inflammatory processes to the onset and progression of Alzheimer’s disease emphasizes the need to identify markers strongly associated with cognitive decline in these patients, and this is crucial for developing strategies to monitor disease progression, stratify risk, and determine the most effective treatment strategies.

## Conclusions

TNF-α and IL-1β appear to play a significant role in cognitive decline in patients with Alzheimer’s disease and mild cognitive impairment, showing potential to distinguish between different stages of Alzheimer’s. Evidence regarding IL-8, IL-6, and IL-10 in AD pathology yields mixed results, highlighting the necessity for further research utilizing cognitive tests that effectively discriminate various aspects of cognitive function assessment. The selection of the blood fraction for analysis appears crucial for obtaining reliable results in inflammatory marker assessments.

## Supplementary Information

Below is the link to the electronic supplementary material.

Supplementary file1 (DOCX 15 KB)—Analysis of levels of IL-8 in the Mild cognitive impairment and control groups. Meta-analysis plot summarizing the effect sizes (with 95% confidence intervals) of levels of IL-8 in MCI and control groups. Each horizontal line represents an individual study, with the square indicating the effect size and the line representing the confidence interval. The square size reflects the study’s weight in the meta-analysis. The diamond at the bottom represents the pooled effect size and its confidence interval.

Supplementary file2 (PNG 41 KB)—Analysis of levels of IL-8 in Alzheimer’s and Mild cognitive impairment. Meta-analysis plot summarizing the effect sizes (with 95% confidence intervals) of levels of IL-8 in Alzheimer’s and Mild cognitive impairment. Each horizontal line represents an individual study, with the square indicating the effect size and the line representing the confidence interval. The square size reflects the study’s weight in the meta-analysis. The diamond at the bottom represents the pooled effect size and its confidence interval.

Supplementary file3 (PNG 75 KB)—Analysis of levels of IL-6 in the Alzheimer’s and control groups. Meta-analysis plot summarizing the effect sizes (with 95% confidence intervals) of levels of IL-6 in Alzheimer’s and control groups. Each horizontal line represents an individual study, with the square indicating the effect size and the line representing the confidence interval. The square size reflects the study’s weight in the meta-analysis. The diamond at the bottom represents the pooled effect size and its confidence interval.

Supplementary file4 (PNG 90 KB)

Supplementary file5 (PNG 61 KB)

Supplementary file6 (PNG 55 KB)

Supplementary file7 (PNG 38 KB)

Supplementary file8 (PNG 62 KB)

Supplementary file9 (PNG 58 KB)

Supplementary file10 (PNG 42 KB)

Supplementary file11 (PNG 34 KB)—Analysis of the MoCA test in the MCI and control groups. Meta-analysis plot summarizing the MoCA test's effect sizes (with 95% confidence intervals) on MCI and control groups. Each horizontal line represents an individual study, with the square indicating the effect size and the line representing the confidence interval. The square size reflects the study's weight in the meta-analysis. The diamond at the bottom represents the pooled effect size and its confidence interval.

Supplementary file12 (PNG 79 KB)—Analysis of levels of IL-1β in Alzheimer's and control groups. Meta-analysis plot summarizing the effect sizes (with 95% confidence intervals) of levels of IL-1B in Alzheimer's and control groups. Each horizontal line represents an individual study, with the square indicating the effect size and the line representing the confidence interval. The square size reflects the study's weight in the meta-analysis. The diamond at the bottom represents the pooled effect size and its confidence interval.

Supplementary file13 (DOCX 22 KB)—Analysis of levels of TNF-α in the Alzheimer's and control groups. Meta-analysis plot summarizing the effect sizes (with 95% confidence intervals) of levels of TNF-α in Alzheimer's and control groups. Each horizontal line represents an individual study, with the square indicating the effect size and the line representing the confidence interval. The square size reflects the study's weight in the meta-analysis. The diamond at the bottom represents the pooled effect size and its confidence interval.

Supplementary file14 (DOCX 20 KB)—Analysis of levels of TNF-α in Mild cognitive impairment (MCI) and control group. Meta-analysis plot summarizing the effect sizes (with 95% confidence intervals) of levels of TNF-α in MCI and control groups. Each horizontal line represents an individual study, with the square indicating the effect size and the line representing the confidence interval. The square size reflects the study's weight in the meta-analysis. The diamond at the bottom represents the pooled effect size and its confidence interval.

Supplementary file15 (DOCX 17 KB)—Analysis of levels of MCP-1 in the Alzheimer's and control groups. Meta-analysis plot summarizing the effect sizes (with 95% confidence intervals) of levels of MCP-1 in MCI and control groups. Each horizontal line represents an individual study, with the square indicating the effect size and the line representing the confidence interval. The square size reflects the study's weight in the meta-analysis. The diamond at the bottom represents the pooled effect size and its confidence interval.

Supplementary file16 (DOCX 16 KB)—Analysis of levels of MCP-1 in the Mild cognitive impairment and control groups. Meta-analysis plot summarizing the effect sizes (with 95% confidence intervals) of levels of MCP-1 in MCI and control groups. Each horizontal line represents an individual study, with the square indicating the effect size and the line representing the confidence interval. The square size reflects the study's weight in the meta-analysis. The diamond at the bottom represents the pooled effect size and its confidence interval.

Supplementary file17 (DOCX 18 KB)—Analysis of levels of IL-10 in the Alzheimer’s and control groups. Meta-analysis plot summarizing the effect sizes (with 95% confidence intervals) of levels of IL-10 in Alzheimer’s and control groups. Each horizontal line represents an individual study, with the square indicating the effect size and the line representing the confidence interval. The square size reflects the study’s weight in the meta-analysis. The diamond at the bottom represents the pooled effect size and its confidence interval.

Supplementary file18 (DOCX 16 KB)—Analysis of levels of IL-8 in the Alzheimer’s and control groups. Meta-analysis plot summarizing the effect sizes (with 95% confidence intervals) of levels of IL-8 in Alzheimer’s and control groups. Each horizontal line represents an individual study, with the square indicating the effect size and the line representing the confidence interval. The square size reflects the study’s weight in the meta-analysis. The diamond at the bottom represents the pooled effect size and its confidence interval.

## Data Availability

The extracted data are available and described in the tables.
