# Supplementary material for: Inflammatory Markers and their Relationship with Cognitive Function in Alzheimer’s Disease and Mild Cognitive Impairment. Systematic Review and Meta-Analysis
Source: Neuromolecular Med. 2025 Jul 25;27(1):53. doi: 10.1007/s12017-025-08866-w (PMC12296862; doi:10.1007/s12017-025-08866-w)
Supplement: Supplementary file 1 — Supplementary file1 (DOCX 15 KB)—Analysis of levels of IL-8 in the Mild cognitive impairment and control groups. Meta-analysis plot summarizing the effect sizes (with 95% confidence intervals) of levels of IL-8 in MCI and control groups. Each horizontal line represents an individual study, with the square indicating the effect size and the line representing the confidence interval. The square size reflects the study’s weight in the meta-analysis. The diamond at the bottom represents the pooled effect size and its confidence interval. [file 12017_2025_8866_MOESM1_ESM.docx]

**APPENDIX No. 1**

**SEARCH STRATEGY**

**MEDLINE (OVID)**

exp Alzheimer's disease OR mild cognitive impairment.mp OR cognitive decline AND ((blood biomarker) or (Peripheral biomarkers)).mp AND inflammation AND exp cohort studies or (cohort* stud*).mp or exp case-control studies or (case*control stud*).mp or exp cross-sectional studies or (cross*sectional stud*).mp

**WEB OF SCIENCE**

((TI= (Alzheimer disease OR mild cognitive impairment OR cognitive decline)) AND AB=(marker* OR blood markers OR biomarker OR peripheral marker)) AND AB=(inflammation OR inflammatory)

**SCOPUS**

(TITLE-ABS-KEY ("Alzheimer disease" OR "mild cognitive impairment" OR “cognitive decline”) AND TITLE-ABS-KEY ("blood biomarker" OR "Peripheral biomarker" OR “biomarker”) AND TITLE-ABS-KEY (“inflammation” OR “inflammatory”) AND "cohort studies" OR "cohort* NEXT2 stud*" OR "case-control studies" OR "case*control NEXT2 stud*" OR "cross-sectional studies" OR "cross*sectional AND NEXT2 stud*"))

**CENTRAL (OVID)**

Alzheimer.mp OR (mild cognitive impairment).mp OR (cognitive decline) AND ("blood biomarker" or "Peripheral biomarkers").mp AND Exp Inflammation or Inflammat*.mp AND exp cohort studies or (cohort* adj2 stud*).mp or exp case-control studies or (case*control adj2 stud*).mp or exp cross-sectional studies or (cross*sectional adj2 stud*).mp

**LILACS**

(ti:(enfermedad de Alzheimer)) OR (ti:deterioro cognitivo leve)) AND (ab:(biomarcador)) AND (ab:(sangre)) AND (ab:(Inflamación)) tw:(casos controles OR transversal OR cohorte)
