# Supplementary figures and images for: Inflammatory Markers and their Relationship with Cognitive Function in Alzheimer’s Disease and Mild Cognitive Impairment. Systematic Review and Meta-Analysis
Source: Neuromolecular Med. 2025 Jul 25;27(1):53. doi: 10.1007/s12017-025-08866-w (PMC12296862; doi:10.1007/s12017-025-08866-w)

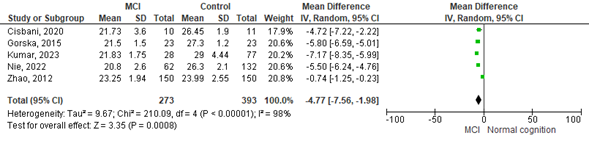

Supplement: Supplementary file 2 — Supplementary file2 (PNG 41 KB)—Analysis of levels of IL-8 in Alzheimer’s and Mild cognitive impairment. Meta-analysis plot summarizing the effect sizes (with 95% confidence intervals) of levels of IL-8 in Alzheimer’s and Mild cognitive impairment. Each horizontal line represents an individual study, with the square indicating the effect size and the line representing the confidence interval. The square size reflects the study’s weight in the meta-analysis. The diamond at the bottom represents the pooled effect size and its confidence interval. [file 12017_2025_8866_MOESM2_ESM.png]

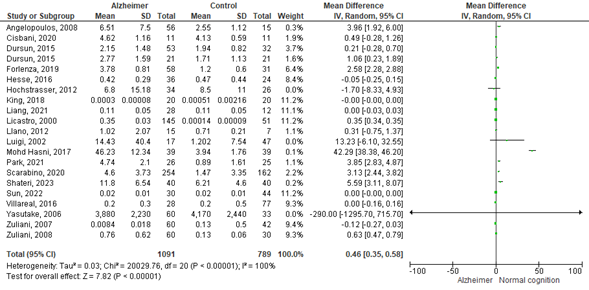

Supplement: Supplementary file 3 — Supplementary file3 (PNG 75 KB)—Analysis of levels of IL-6 in the Alzheimer’s and control groups. Meta-analysis plot summarizing the effect sizes (with 95% confidence intervals) of levels of IL-6 in Alzheimer’s and control groups. Each horizontal line represents an individual study, with the square indicating the effect size and the line representing the confidence interval. The square size reflects the study’s weight in the meta-analysis. The diamond at the bottom represents the pooled effect size and its confidence interval. [file 12017_2025_8866_MOESM3_ESM.png]

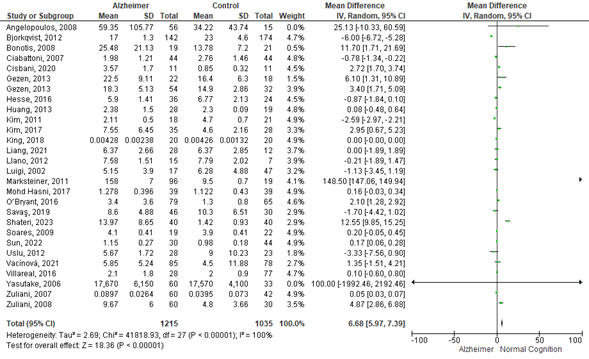

Supplement: Supplementary file 4 — Supplementary file4 (PNG 90 KB) [file 12017_2025_8866_MOESM4_ESM.png]

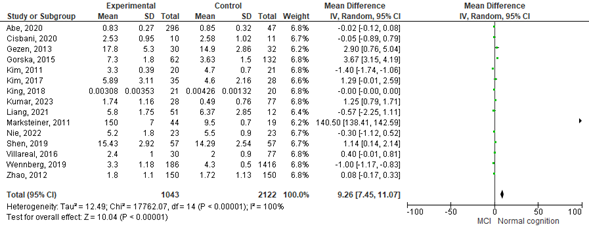

Supplement: Supplementary file 5 — Supplementary file5 (PNG 61 KB) [file 12017_2025_8866_MOESM5_ESM.png]

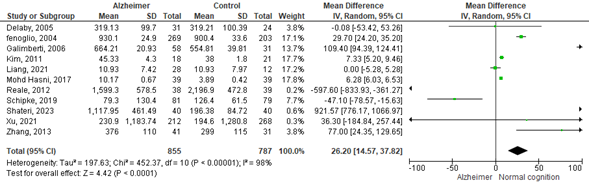

Supplement: Supplementary file 6 — Supplementary file6 (PNG 55 KB) [file 12017_2025_8866_MOESM6_ESM.png]

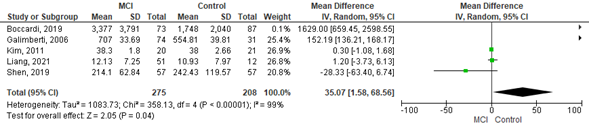

Supplement: Supplementary file 7 — Supplementary file7 (PNG 38 KB) [file 12017_2025_8866_MOESM7_ESM.png]

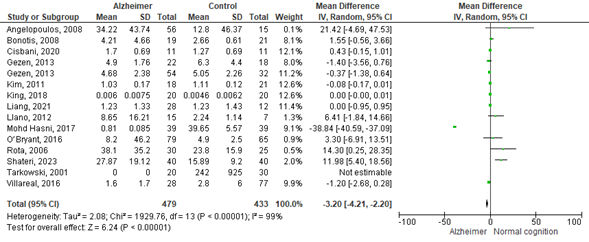

Supplement: Supplementary file 8 — Supplementary file8 (PNG 62 KB) [file 12017_2025_8866_MOESM8_ESM.png]

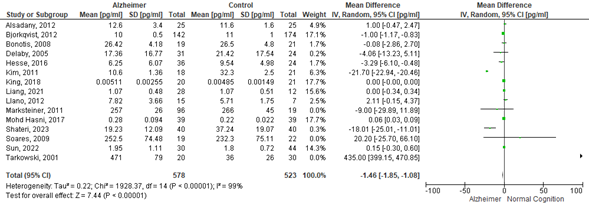

Supplement: Supplementary file 9 — Supplementary file9 (PNG 58 KB) [file 12017_2025_8866_MOESM9_ESM.png]

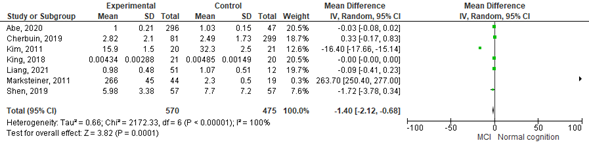

Supplement: Supplementary file 10 — Supplementary file10 (PNG 42 KB) [file 12017_2025_8866_MOESM10_ESM.png]

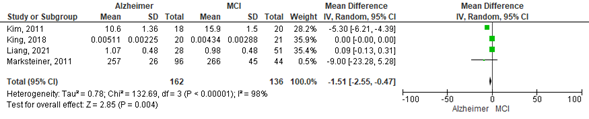

Supplement: Supplementary file 11 — Supplementary file11 (PNG 34 KB)—Analysis of the MoCA test in the MCI and control groups. Meta-analysis plot summarizing the MoCA test's effect sizes (with 95% confidence intervals) on MCI and control groups. Each horizontal line represents an individual study, with the square indicating the effect size and the line representing the confidence interval. The square size reflects the study's weight in the meta-analysis. The diamond at the bottom represents the pooled effect size and its confidence interval. [file 12017_2025_8866_MOESM11_ESM.png]

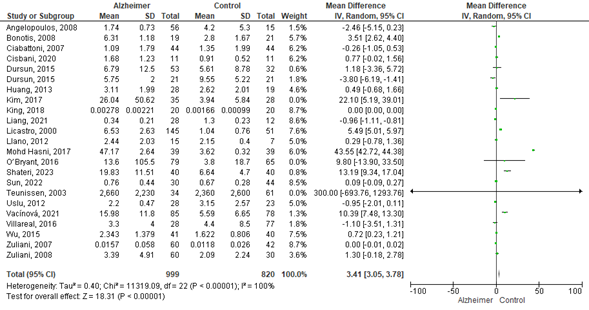

Supplement: Supplementary file 12 — Supplementary file12 (PNG 79 KB)—Analysis of levels of IL-1β in Alzheimer's and control groups. Meta-analysis plot summarizing the effect sizes (with 95% confidence intervals) of levels of IL-1B in Alzheimer's and control groups. Each horizontal line represents an individual study, with the square indicating the effect size and the line representing the confidence interval. The square size reflects the study's weight in the meta-analysis. The diamond at the bottom represents the pooled effect size and its confidence interval. [file 12017_2025_8866_MOESM12_ESM.png]
