# Supplementary material for: Inflammatory Markers and their Relationship with Cognitive Function in Alzheimer’s Disease and Mild Cognitive Impairment. Systematic Review and Meta-Analysis
Source: Neuromolecular Med. 2025 Jul 25;27(1):53. doi: 10.1007/s12017-025-08866-w (PMC12296862; doi:10.1007/s12017-025-08866-w)
Supplement: Supplementary file 13 — Supplementary file13 (DOCX 22 KB)—Analysis of levels of TNF-α in the Alzheimer's and control groups. Meta-analysis plot summarizing the effect sizes (with 95% confidence intervals) of levels of TNF-α in Alzheimer's and control groups. Each horizontal line represents an individual study, with the square indicating the effect size and the line representing the confidence interval. The square size reflects the study's weight in the meta-analysis. The diamond at the bottom represents the pooled effect size and its confidence interval. [file 12017_2025_8866_MOESM13_ESM.docx]

| First author & reference | Selection | | | | Comparability | Exposure | | Score |
| --- | --- | --- | --- | --- | --- | --- | --- | --- |
|  | Is the Case Definition Adequate? | Representativen ess of the Cases | Selection of Controls | Definition of Controls | Comparability of cases and controls | Ascertainment of exposure | Same method of ascertainment for cases and controls | Total |
| Boccardi, 2019 | 1 | 1 | 1 | 1 | 1 | 1 | 1 | 7 |
| Cherbuin, 2019 | 1 | 1 | 1 | 0 | 1 | 1 | 1 | 6 |
| Cisbani, 2020 | 1 | 1 | 1 | 1 | 1 | 1 | 1 | 7 |
| Delaby, 2005 | 1 | 1 | 0 | 0 | 1 | 1 | 1 | 5 |
| Kim, 2017 | 1 | 1 | 1 | 0 | 1 | 1 | 1 | 6 |
| Koca, 2022 | 1 | 1 | 1 | 1 | 1 | 1 | 1 | 7 |
| MohdHasni, 2017 | 1 | 1 | 1 | 1 | 1 | 1 | 1 | 7 |
| Nie, 2022 | 1 | 1 | 1 | 1 | 1 | 1 | 1 | 7 |
| Savaş, 2019 | 1 | 1 | 1 | 1 | 1 | 1 | 1 | 7 |
| Scarabino, 2020 | 1 | 1 | 1 | 0 | 0 | 1 | 1 | 5 |
| Shateri, 2023 | 1 | 0 | 1 | 1 | 1 | 1 | 1 | 6 |
| Startin, 2019 | 1 | 0 | 1 | 1 | 1 | 1 | 1 | 6 |
| Vacínová, 2021 | 1 | 1 | 1 | 1 | 0 | 1 | 1 | 6 |
| Zhao, 2012 | 1 | 1 | 1 | 1 | 1 | 1 | 1 | 7 |
| Dursun, 2015 | 1 | 1 | 1 | 1 | 2 | 1 | 1 | 8 |
| Luigi, 2002 | 1 | 1 | 1 | 0 | 1 | 1 | 1 | 6 |
| Rota, 2006 | 1 | 1 | 1 | 1 | 1 | 1 | 1 | 7 |
| Linberg, 2005 | 1 | 0 | 1 | 1 | 0 | 1 | 1 | 5 |
| Teunissen, 2003 | 1 | 1 | 1 | 0 | 1 | 1 | 1 | 6 |
| Zaciragic, 2007 | 1 | 1 | 1 | 1 | 1 | 1 | 1 | 7 |
| Zuliani, 2008 | 1 | 1 | 1 | 1 | 1 | 1 | 1 | 7 |
| Zuliani, 2007 | 1 | 1 | 1 | 1 | 1 | 1 | 1 | 7 |
| Licastro, 2000 | 1 | 1 | 1 | 1 | 1 | 1 | 1 | 7 |
| Forlenza, 2019 | 1 | 0 | 0 | 0 | 1 | 1 | 1 | 4 |
| Malaguarnera, 2006 | 1 | 0 | 1 | 1 | 1 | 1 | 1 | 6 |
| Motta, 2007 | 1 | 0 | 1 | 1 | 1 | 1 | 1 | 6 |
| Tarkowski, 2001 | 1 | 1 | 1 | 1 | 1 | 1 | 1 | 7 |
| Fenoglio, 2004 | 1 | 1 | 1 | 1 | 2 | 1 | 1 | 8 |
| Gongora, 2020 | 1 | 1 | 1 | 1 | 1 | 1 | 1 | 7 |
| Hesse, 2016 | 1 | 0 | 1 | 1 | 2 | 1 | 1 | 7 |
| Kim, 2011 | 1 | 1 | 1 | 1 | 2 | 1 | 1 | 8 |
| laske, 2008 | 1 | 0 | 1 | 0 | 2 | 1 | 1 | 6 |
| llano, 2012 | 1 | 1 | 1 | 1 | 1 | 1 | 1 | 7 |
| Marksteiner, 2011 | 0 | 0 | 0 | 0 | 1 | 1 | 1 | 3 |
| O’Bryant, 2016 | 1 | 0 | 1 | 1 | 1 | 1 | 1 | 6 |
| Perea, 2018 | 1 | 0 | 1 | 1 | 1 | 1 | 1 | 6 |
| Reale, 2012 | 1 | 1 | 0 | 1 | 1 | 1 | 1 | 6 |
| Schipke, 2019 | 1 | 1 | 0 | 0 | 0 | 1 | 1 | 4 |
| Soares, 2009 | 1 | 0 | 0 | 0 | 1 | 1 | 1 | 4 |
| Wu, 2015 | 1 | 0 | 0 | 1 | 0 | 1 | 1 | 4 |
| Xu, 2021 | 1 | 1 | 1 | 0 | 2 | 1 | 1 | 7 |
| Zhang, 2013 | 1 | 1 | 1 | 1 | 1 | 1 | 1 | 7 |
| Galimberti, 2006 | 1 | 1 | 1 | 1 | 1 | 1 | 1 | 7 |
| Hochstrasser, 2012 | 1 | 0 | 1 | 1 | 1 | 1 | 1 | 6 |
| Shen, 2019 | 1 | 1 | 1 | 1 | 1 | 1 | 1 | 7 |
| Dubenko , 2021 | 1 | 1 | 1 | 1 | 1 | 1 | 1 | 7 |
| Ramadan , 2022 | 1 | 0 | 1 | 0 | 0 | 1 | 1 | 4 |
| Gezen , 2013 | 1 | 0 | 1 | 0 | 1 | 1 | 1 | 5 |
