# Supplementary material for: Inflammatory Markers and their Relationship with Cognitive Function in Alzheimer’s Disease and Mild Cognitive Impairment. Systematic Review and Meta-Analysis
Source: Neuromolecular Med. 2025 Jul 25;27(1):53. doi: 10.1007/s12017-025-08866-w (PMC12296862; doi:10.1007/s12017-025-08866-w)
Supplement: Supplementary file 14 — Supplementary file14 (DOCX 20 KB)—Analysis of levels of TNF-α in Mild cognitive impairment (MCI) and control group. Meta-analysis plot summarizing the effect sizes (with 95% confidence intervals) of levels of TNF-α in MCI and control groups. Each horizontal line represents an individual study, with the square indicating the effect size and the line representing the confidence interval. The square size reflects the study's weight in the meta-analysis. The diamond at the bottom represents the pooled effect size and its confidence interval. [file 12017_2025_8866_MOESM14_ESM.docx]

| First author & reference | Selection | | | | Comparability | Exposure | | | Score |  |
| --- | --- | --- | --- | --- | --- | --- | --- | --- | --- | --- |
|  | Is the eligibility criteria specified? | Representativeness of the sample | Selection of sample/sample size | Definition of no included subjects | Comparability between participants | Assessment of outcomes | Same method of Assessment of outcome for all the sample | Statistical test | Total |  |
|  |  |  |  |  |  |  |  |  |  |  |
| Huang, 2013 | 1 | 1 | 1 | 1 | 1 | 1 | 1 | 1 | 8 |  |
| Julian, 2015 | 1 | 1 | 1 | 1 | 1 | 1 | 1 | 1 | 8 |  |
| King, 2018 | 1 | 1 | 1 | 1 | 1 | 1 | 1 | 1 | 8 |  |
| Mahdavi, 2021 | 1 | 1 | 1 | 1 | 1 | 1 | 1 | 1 | 8 |  |
| Marzabadi, 2021 | 1 | 1 | 1 | 1 | 1 | 1 | 1 | 1 | 8 |  |
| Park, 2021 | 1 | 1 | 1 | 1 | 1 | 1 | 1 | 1 | 8 |  |
| Richartz, 2005 | 0 | 1 | 0 | 0 | 1 | 1 | 1 | 1 | 5 |  |
| Sun, 2003 | 0 | 1 | 0 | 0 | 1 | 1 | 1 | 1 | 5 |  |
| Sun, 2022 | 1 | 1 | 1 | 1 | 1 | 1 | 1 | 1 | 8 |  |
| Uslu, 2012 | 1 | 1 | 0 | 0 | 1 | 1 | 1 | 1 | 6 |  |
| Villareal, 2016 | 1 | 1 | 0 | 1 | 1 | 1 | 1 | 1 | 7 |  |
| Kumar, 2023 | 1 | 1 | 1 | 0 | 1 | 1 | 1 | 1 | 7 |  |
| Paganelli, 2002 | 1 | 1 | 1 | 0 | 1 | 1 | 1 | 1 | 7 |  |
| Ciabattoni, 2007 | 1 | 1 | 0 | 0 | 1 | 1 | 1 | 1 | 6 |  |
| Angelopoulos, 2008 | 0 | 1 | 0 | 0 | 1 | 1 | 1 | 1 | 5 |  |
| Bonotis, 2008 | 0 | 1 | 0 | 0 | 1 | 1 | 1 | 1 | 5 |  |
| Yasutake, 2006 | 1 | 1 | 1 | 0 | 1 | 1 | 1 | 1 | 7 |  |
| Lwalor, 1996 | 1 | 1 | 0 | 1 | 1 | 1 | 1 | 1 | 7 |  |
| Bossu, 2008 | 1 | 1 | 1 | 1 | 1 | 1 | 1 | 1 | 8 |  |
| Alsadany, 2012 | 1 | 1 | 1 | 1 | 1 | 1 | 1 | 1 | 8 |  |
| Bjorkqvist, 2012 | 1 | 1 | 1 | 0 | 1 | 1 | 1 | 1 | 7 |  |
| Kim, 2008 | 1 | 1 | 1 | 1 | 1 | 1 | 1 | 1 | 8 |  |
| King , 2019 | 1 | 1 | 1 | 1 | 1 | 1 | 1 | 1 | 8 |  |
| Culjak , 2020 | 1 | 1 | 1 | 1 | 1 | 1 | 1 | 1 | 8 |  |
| Gorska , 2015 | 1 | 1 | 1 | 1 | 1 | 1 | 1 | 1 | 8 |  |
