# Supplementary material for: Inflammatory Markers and their Relationship with Cognitive Function in Alzheimer’s Disease and Mild Cognitive Impairment. Systematic Review and Meta-Analysis
Source: Neuromolecular Med. 2025 Jul 25;27(1):53. doi: 10.1007/s12017-025-08866-w (PMC12296862; doi:10.1007/s12017-025-08866-w)
Supplement: Supplementary file 15 — Supplementary file15 (DOCX 17 KB)—Analysis of levels of MCP-1 in the Alzheimer's and control groups. Meta-analysis plot summarizing the effect sizes (with 95% confidence intervals) of levels of MCP-1 in MCI and control groups. Each horizontal line represents an individual study, with the square indicating the effect size and the line representing the confidence interval. The square size reflects the study's weight in the meta-analysis. The diamond at the bottom represents the pooled effect size and its confidence interval. [file 12017_2025_8866_MOESM15_ESM.docx]

| First author & reference | Selection | | | | Comparability | Exposure | | | Score |  |
| --- | --- | --- | --- | --- | --- | --- | --- | --- | --- | --- |
|  | Representativeness of the exposed cohort | Selection of the non exposed cohort | Ascertainment of exposure | Demonstration that outcome of interest was not present at start of study | Comparability of cohorts on the basis of the design or analysis -2 | Assessment of outcome | Was follow-up long enough for outcomes to occur | Adequacy of follow up of cohorts | Total |  |
|  |  |  |  |  |  |  |  |  |  |  |
| Abe, 2020 | 1 | 1 | 1 | 1 | 2 | 1 | 0 | 0 | 7 |  |
| Gross, 2019 | 1 | 1 | 1 | 1 | 2 | 1 | 1 | 1 | 8 |  |
| Karim, 2014 | 1 | 0 | 1 | 0 | 0 | 1 | 0 | 1 | 3 |  |
| Leung, 2013 | 1 | 1 | 1 | 1 | 1 | 1 | 0 | 1 | 6 |  |
| Liang, 2021 | 1 | 1 | 1 | 1 | 1 | 1 | 1 | 1 | 7 |  |
| Wennberg, 2019 | 1 | 1 | 1 | 1 | 1 | 1 | 0 | 1 | 6 |  |
| Gupta, 2017 | 1 | 1 | 1 | 1 | 1 | 1 | 1 | 1 | 7 |  |
| Hazen, 2019 | 1 | 0 | 1 | 1 | 1 | 1 | 1 | 1 | 6 |  |
| Lee, 2018 | 1 | 1 | 1 | 1 | 1 | 1 | 1 | 1 | 7 |  |
| Westin, 2012 | 1 | 1 | 1 | 1 | 1 | 1 | 1 | 1 | 7 |  |
| Holmes, 2011 | 1 | 1 | 1 | 0 | 1 | 1 | 1 | 1 | 6 |  |
