# Supplementary material for: Inflammatory Markers and their Relationship with Cognitive Function in Alzheimer’s Disease and Mild Cognitive Impairment. Systematic Review and Meta-Analysis
Source: Neuromolecular Med. 2025 Jul 25;27(1):53. doi: 10.1007/s12017-025-08866-w (PMC12296862; doi:10.1007/s12017-025-08866-w)
Supplement: Supplementary file 16 — Supplementary file16 (DOCX 16 KB)—Analysis of levels of MCP-1 in the Mild cognitive impairment and control groups. Meta-analysis plot summarizing the effect sizes (with 95% confidence intervals) of levels of MCP-1 in MCI and control groups. Each horizontal line represents an individual study, with the square indicating the effect size and the line representing the confidence interval. The square size reflects the study's weight in the meta-analysis. The diamond at the bottom represents the pooled effect size and its confidence interval. [file 12017_2025_8866_MOESM16_ESM.docx]

| **TEST** | **# studies**  **included** | **N (P/NC)** | **Main Effect** | | | | **Heterogeneity** | | | **Difference score** |
| --- | --- | --- | --- | --- | --- | --- | --- | --- | --- | --- |
|  |  |  | **MD** | **95% CI** | **z** | ***p*** | **Chi^2^** | ***p*** | **I^2^** |  |
| **MMSE (AD/NC)** | 49 | 2902/2838 | -10,21 | -11,03, -9,38 | 24,24 | <0.00001 | 4666,56 | <0.00001 | 99 | **↓** |
| **MMSE (MCI/NC)** | 26 | 1580/2016 | -2,72 | -3,30, -2,14 | 9,25 | <0.00001 | 769,20 | <0.00001 | 100 | **↓** |
| **MMSE (AD/MCI)** | 20 | 1393/1011 | -7,58 | -9,03, -6,13 | 10,23 | <0.00001 | 1597,77 | <0.00001 | 99 | **↓** |
| MMSE: Mini mental test; AD: Alzheimer; MCI: Mild cognitive impairment; NC: Normal cognition; P: grouped sample of the studies; MD: mean difference | | | | | | | | | | |
