# Supplementary material for: Inflammatory Markers and their Relationship with Cognitive Function in Alzheimer’s Disease and Mild Cognitive Impairment. Systematic Review and Meta-Analysis
Source: Neuromolecular Med. 2025 Jul 25;27(1):53. doi: 10.1007/s12017-025-08866-w (PMC12296862; doi:10.1007/s12017-025-08866-w)
Supplement: Supplementary file 17 — Supplementary file17 (DOCX 18 KB)—Analysis of levels of IL-10 in the Alzheimer’s and control groups. Meta-analysis plot summarizing the effect sizes (with 95% confidence intervals) of levels of IL-10 in Alzheimer’s and control groups. Each horizontal line represents an individual study, with the square indicating the effect size and the line representing the confidence interval. The square size reflects the study’s weight in the meta-analysis. The diamond at the bottom represents the pooled effect size and its confidence interval. [file 12017_2025_8866_MOESM17_ESM.docx]

| **Biomarker** | **# studies**  **included** | **n** | **Main Effect** | | | | **Heterogeneity** | | | **Regulation described** |
| --- | --- | --- | --- | --- | --- | --- | --- | --- | --- | --- |
|  |  | **AD/NC** | **MD** | **95% CI** | **z** | ***p*** | **Chi^2^** | ***p*** | **I^2^** |  |
| **IL-8 (serum)** | 5 | 145/138 | 43,40 | 27,83, 58,97 | 5,46 | <0.00001 | 571,64 | <0.00001 | 99 | **↑** |
| **IL-8 (plasma)** | 10 | 316/385 | -3,43 | -5,13, -1,73 | 3,96 | <0.00001 | 1272,13 | <0.00001 | 99 | **↓** |
| **TNF-α (plasma)** | 18 | 767/761 | 8,66 | 7,83, 9,49 | 20,45 | <0.00001 | 41691,9 | <0.00001 | 100 | **↑** |
| **TNF-α (serum)** | 10 | 409/343 | 0,93 | -0,3, 1,9 | 1,89 | 0,06 | 62,57 | <0.00001 | 86 | **↑** |
| **IL-1β (serum)** | 8 | 362/283 | 3,70 | 2,37, 5,03 | 5,45 | <0.00001 | 706,92 | <0.00001 | 99 | **↑** |
| **IL-1β (plasma)** | 12 | 691/711 | 9,01 | 8,15, 9,87 | 20,50 | <0.00001 | 4699, 90 | <0.00001 | 100 | **↑** |
| **Il-6 (plasma)** | 14 | 702/519 | 0,31 | 0,16, 046 | 4,05 | <0.00001 | 776,66 | <0.00001 | 98 | **↑** |
| **IL-6 (serum)** | 9 | 375/370 | 8,01 | -7,08, 23,10 | 1,04 | 0,30 | 6968,61 | <0.00001 | 100 | **↑** |
| **MCP-1 (serum)** | 6 | 690/644 | 24,23 | -3,51, 51,97 | 1,71 | 0,09 | 261,81 | <0.00001 | 98 | **↑** |
| **MCP-1 (plasma)** | 5 | 165/143 | 48,84 | 15,47, 82,22 | 2,87 | 0,004 | 190,53 | <0.00001 | 98 | **↑** |
| AD: Alzheimer disease; MCI: Mild cognitive impairment; MD: mean difference; IL: interleukin; TNF: Tumoral necrosis factor; MCP- 1: monocyte chemoattractant protein 1 | | | | | | | | | | |
